# Health-State Utility Values in CP Patients Following Deformity Surgery: Are We Now Ready for Cost-Utility Analysis in This Patient Population?

**DOI:** 10.3390/jcm15093398

**Published:** 2026-04-29

**Authors:** Firoz Miyanji, Luigi A. Nasto, Amer Samdani, Suken A. Shah, Unni G. Narayanan, Amit Jain, Tracey P. Bryan, Peter O. Newton, Paul D. Sponseller

**Affiliations:** 1 British Columbia Children’s Hospital, 4500 Oak St, Vancouver, BC V6H 3N1, Canada; luigianasto@gmail.com; 2 Shriners Children’s Philadelphia, 3551 N Broad St, Philadelphia, PA 19140, USA; asamdani@shrinenet.org; 3 Nemours Children’s Hospital, 1600 Rockland Rd, Wilmington, DE 19803, USA; suken.shah@nemours.org; 4 The Hospital for Sick Children, 170 Elizabeth St, Toronto, ON M5G 1E8, Canada; unni.narayanan@sickkids.ca; 5 The Johns Hopkins Hospital, 1800 Orleans St, Baltimore, MD 21287, USA; amitjain@jhmi.edu (A.J.); psponse@gmail.com (P.D.S.); 6 Rady Children’s Hospital, 3020 Children’s Way, San Diego, CA 92123, USA; tbryan@rchsd.org (T.P.B.); pnewton.rady@gmail.com (P.O.N.)

**Keywords:** cost-utility analysis, CPCHILD, HUI3, cerebral palsy, neuromuscular scoliosis, mapping algorithm

## Abstract

**Background:** Cost-utility analysis (CUA) is frequently used by reimbursement agencies and national advisory bodies to make informed decisions on whether or not to reimburse surgical interventions. Health state preferences (utilities) are a key component in valuing health outcomes in that they are used in calculating quality-adjusted life-years (QALY). Unfortunately, disease-specific HRQoL measures commonly lack the preference weights necessary to produce health-state utility values for use in CUA. A solution to this problem is to map a disease-specific quality-of-life measure to a generic preference-based measure. The aim of this study was to develop health-state utility values for cerebral palsy (CP) patients with scoliosis by mapping disease-specific quality-of-life scores (CPCHILD outcome questionnaire) to the Health Utility Index Mark 3 (HUI3) questionnaire. **Methods:** A prospective, multicentre CP scoliosis database was analysed identifying consecutive CP patients with ≥2 years follow-up who completed both the CPCHILD and HUI3 at enrolment, at 1-, and at 2 years follow-up. Ordinary least squared regression models were constructed to estimate HUI3 utility values from CPCHILD scores and clinical variables. The model was developed using enrolment data, while 1- and 2-years follow-up data were used for confirmatory analysis of the goodness of fit of the model (i.e., paired *t* test between observed and calculated HUI utility values). **Results:** A total of 232 patients were included, 91.9% were GMFCS IV and V, 87.9% underwent surgery during the study period, and the average magnitude of scoliosis deformity at enrolment was 81.93° ± 25.13°. A log-linear regression model was developed, including three predicting variables: CPCHILD total score (β = 0.016, *p* = 0.0001), communication (β = −0.436, *p* = 0.0001), and feeding ability (β = −0.289, *p* = 0.0001). The R^2^ of the model was 0.578, and *F* 49.73 (*p* = 0.0001). The mean difference of means between observed HUI3 values and calculated HUI3 values at 1- and 2 years was −0.020 (*p =* 0.129) and 0.017 points (*p =* 0.187), respectively. **Conclusions:** Although the use of a preference-based HRQoL measure is the ideal method to generate health-state utility values, we demonstrate that HUI3 scores can be accurately predicted using the CPCHILD questionnaire. This mapping algorithm will be useful in estimating health-state utilities in clinical trials, and hence CUA, of CP patients undergoing scoliosis surgery to help better inform patients, care-givers, health-care providers, and decision makers of the economic burden of surgery in this patient population.

## 1. Introduction

As healthcare costs continue to increase, more and more attention is being placed on resources allocation and value provided by different health interventions. Cost-utility analysis (CUA) is a form of economic evaluation frequently used by reimbursement agencies and national advisory bodies to measure and compare value of health interventions in terms of improved quality and quantity of life [1]. While measuring a person’s duration of life is relatively easy, this is not the case for a highly subjective measure like health related quality of life (HRQoL). CUA makes use of validated, preference based, generic questionnaires specifically designed to measure HRQoL and assign it to a utility value on a scale from 0.0 (dead) to 1.0 (perfect health) [2]. Once a utility value is obtained this is multiplied by the number of years in that health status to generate quality-adjusted life years (QALY). QALY is the primary outcome measure of CUA and allows direct comparison of value provided by different health interventions [3]. Patients with Cerebral Palsy often present with significant spinal deformity requiring surgical intervention, placing a high financial demand on families and healthcare systems. QALYs can help determine if the high-cost of surgical treatment offers a proportional improvement in the patient’s quality of life.

Measurement of health state utility values (HSUV) is a key component of QALY and CUA analysis. Examples of validated tools for HSUV generation include the Health Utility Index Mark 3 (HUI3), the Short-Form 6 dimensions (SF-6D), and the EuroQol 5 dimensions (EQ-5D) questionnaires [2]. The utility values for these instruments are based upon surveys of the general population and are fundamentally different from disease specific HRQoL questionnaires routinely used in paediatric spinal surgery practice (e.g., SRS-22r, EOSQ-24, or CHQ). Disease specific tools lack preference weights and reference to population norms that are the main characteristics of HSUV generating tools. As a result, most of the currently available evidence in paediatric spinal surgery is not amenable for CUA evaluation [4]. A potential solution to this problem is to map an already available and validated disease-specific questionnaire to a generic preference-based tool, such as HUI3. Examples of this approach are available for several conditions [5,6,7,8], but no similar attempts have been made for neuromuscular scoliosis.

The Caregiver Priorities and Child Health Index of Life with Disabilities (CPCHILD^TM^) is a disease-specific, caregiver proxy, measure of health status and well-being of children with severe cerebral palsy (CP) [9]. CPCHILD has been validated for use in CP patients and it has been adopted by many high-volume paediatric spinal surgery centres. Several recent reports in spinal literature have used CPCHILD and have shown positive results of scoliosis surgery in CP patients at short- and medium-term follow-up [10,11,12]. In this manuscript, we present a mapping algorithm for conversion of CPCHILD outcome data into HUI3 utility values for patients with scoliosis and CP. The aim of this study is to allow researchers to be able to convert their outcome data into HUI3 utility values for future CUA evaluation of scoliosis surgery in CP patients.

## 2. Materials and Methods

Following Institutional Review Board (IRB) approval, we retrospectively reviewed all consecutive cases of neuromuscular scoliosis enrolled in a prospective, longitudinal, multi-centre database from June 2008 to September 2014. All patients had a diagnosis of cerebral palsy (CP), patients with other neuromuscular spinal deformities were excluded. The database consisted of surgical (87.5%) and non-surgical (12.5%) patients. Patients who received surgery for their deformity underwent an instrumented fusion (posterior or combined anterior and posterior), while non-surgical patients were managed by orthosis, wheelchair adjustment, physiotherapy, and pain medications as needed during their follow-up. Additional inclusion criteria for the study were: (1) major Cobb angle ≥ 40° (either scoliosis or kyphosis); (3) age 8–21 years, (4) follow-up ≥ 2 years. Patients’ caregivers signed a consent form at the enrolment into the database. All data were collected by independent researchers not involved in data analysis or clinical care of the patients. Primary outcome measures of the study were the paired CPCHILD and HUI3 scores collected at the enrolment (or pre-operative for surgical patients), at 1-, and at 2-years follow-up. General demographics, comorbidities, and deformity radiographical data were also collected.

### 2.1. Study Instruments

CPCHILD Questionnaire. The Child Health Index of Life with Disabilities (CPCHILD^TM^) questionnaire is a validated, caregiver proxy, disease-specific instrument designed for measuring HRQoL in children with CP. This instrument consists of 37 item questions across 6 domains: (1) personal care and activities of daily living, (2) positioning, transferring, and mobility, (3) comfort and emotions, (4) communication and social interaction, (5) general health, (6) overall quality of life. All 6 items are scored separately on a 0 (worst outcome) to 100 (best outcome) points scale, or combined in a single total score (0 to 100 points) [9].

HUI3 Questionnaire. The Health Utility Index (HUI) Mark 3 is a generic, preference-based, comprehensive system for measuring health status and HRQoL and for producing utility scores. HUI3 consists of 8 attributes or domains: (1) vision, (2) hearing, (3) speech, (4) ambulation, (5) dexterity, (6) emotion, (7) cognition, and (8) pain. Each attribute is scored on a 5 or 6 levels scale and a coding algorithm is used to produce utility scores (between 0.000 and 1.000) for each attribute. Finally, attributes scores are combined in a total HRQoL utility score defined on a 0.000 (dead) to 1.000 (perfect health) scale. The HUI3 also allows negative HRQoL total scores to account for health status considered worse than dead do; the lowest possible score for HUI3 is −0.360 [13].

### 2.2. Additional Measures

General demographic data were collected for all patients, including comorbidities as detailed in patients’ charts. Standard sitting antero-posterior (AP) and lateral views X-rays of the whole spine were obtained in all patients. Radiographic data included antero-posterior Cobb angle, pelvic obliquity, thoracic kyphosis, and lumbar lordosis angles.

### 2.3. Statistical Analysis

Descriptive statistics were used to examine characteristics of the study population. Data were presented as average ± standard deviation (range); counts and percentages were used as appropriate. Data normal distribution was checked by histogram analysis, normal probability q-q plots, and Shapiro-Wilk test. Correlation between paired CPCHILD and HUI3 domain scores was assessed by Spearman’s *ρ* coefficients. Using enrolment data, ordinary least squared (OLS) regression models were constructed to estimate HUI3 utility scores from CPCHILD domain scores. Demographic and clinical variables (e.g., age, sex, GMFCS level, and curve magnitude) were also added to the models. Plots of the residuals were examined in each model and quadratic terms for CPCHILD scores were tested if nonlinearity was detected. Fit and predictive accuracy of the models was compared using *F* test, root mean square error (RMSE), and adjusted R^2^ statistics. Follow-up data at 1- and 2-years were used to test predictive accuracy of the final model. Predicted HUI3 scores at 1- and 2-years follow-up were calculated and paired *t*-test was used to compare means of the observed and estimated HUI3 scores. All tests were two-tailed and *p* values ≤ 0.05 were considered significant. Data were analyzed using IBM SPSS Statistics (version 21.0; IBM, Armonk, NY, USA).

## 3. Results

**Figure 1:**
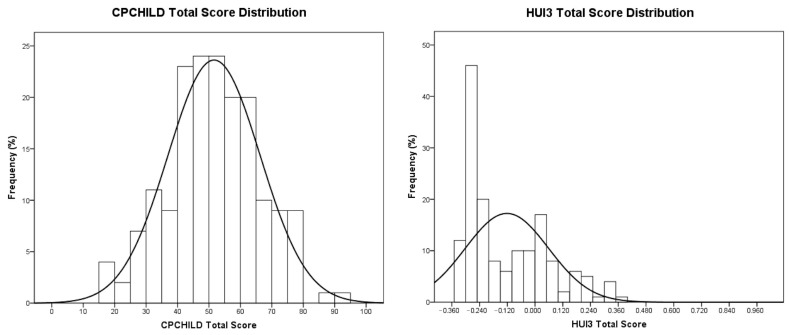
Histogram distribution of the CPCHILD Total Scores (**left panel**) and HUI3 Total Utility Values (**right panel**). Normal curve is shown in both graphs demonstrating a left skewed distribution of the HUI3 scores (**right panel**).

The demographics and clinical characteristics of the study population are presented in Table 1. A total of 232 patients (126 males, average age 14.05 ± 2.60 years) were included. Most of the patients (91.9%) were classified as GMFCS level IV and V, and the average Cobb angle was 81.93° ± 25.13°. This is consistent with the reported higher prevalence of spinal deformity in more severely affected CP patients. Significant developmental delay (77.1%) and communication impairment (72.6%) were also very common in our cohort. CPCHILD and HUI3 scores at the enrolment are shown in Table 2 and Table 3. Average CPCHILD total score was 51.87 ± 14.56 and scores were normally distributed (*p* = 0.599, *skewness* −0.021). The average HUI3 total score was −0.096 ± 0.222 but histogram analysis confirmed a skewed distribution of the scores to the left (i.e., lower scores, *skewness* 0.823), which is consistent with the larger number of severely affected GMFCS level IV and V patients included. (Figure 1).

In order for CPCHILD to be able to predict HUI3 utility values there must be some overlap and correlation between domains measured by the two questionnaires. As shown in Table 4, HUI3 total utility score correlated significantly with all CPCHILD domains, with the only exception of the personal care domain. Strongest correlation was observed between HUI3 total and CPCHILD communication score (*ρ* = 0.646, *p* = 0.00001), HUI3 pain and CPCHILD comfort score (*ρ* = 0.644, *p* = 0.00001), HUI3 cognition and speech attributes and CPCHILD communication score (*ρ* = 0.585 and *ρ* = 0.556, respectively, *p* = 0.00001), and HUI3 pain and CPCHILD total score (*ρ* = 0.530, *p* = 0.00001). The only exceptions were the HUI3 hearing attribute which did not correlate with any CPCHILD domain, and the CPCHILD personal domain which weakly correlated with the HUI3 pain attribute only (*ρ* = 0.276, *p* = 0.00002).

### 3.1. Predictive Model

Because of the skewed distribution of the HUI3 total scores (dependent variable), a natural logarithm transformation was applied to the dependent variable to construct a log-linear regression model. Table 5 shows the final model used for HUI3 total utility value prediction (*F* = 49.73, *p* = 0.0001, adjusted R^2^ = 0.547). The variables included in the model were CPCHILD total score (*t* = 3.98, *p* = 0.0001), ability to communicate (*t* = −5.86, *p* = 0.0001), and feeding (*t* = −4.28, *p* = 0.0001). To obtain the predicted HUI3 utility score, natural logarithm of the HUI3 score is calculated first:

$$\ln\left(HUI3\right)=\;-1.700+\left(0.016\cdot CPCHIL\;total\right)-\;\left(0.436\;\cdot Communication\right)-\;(0.289\cdot Feeding)$$

where *communication* is coded as fully verbal (score 0), limited verbal (score 1), non-verbal (score 2) and *feeding* is coded as oral only (score 0), oral and G-tube (score 1), G-tube only (score 2).

Then, the value obtained is used to compute the final HUI3 utility value:

$$HUI3=\;{(e}^{ln(HUI3)}\cdot 1.195)-0.360$$

### 3.2. Confirmatory Analysis

The results of the confirmatory analysis are presented in Table 6. The model was used to predict HUI3 utility values at 1- and 2-years follow-up using CPCHILD scores at the same time points. There was no significant difference between observed and calculated HUI3 utility values at 1 year (0.020, 95% CI −0.045–0.006, *p* = 0.129). Similarly, no significant difference was noted between calculated and observed values at 2 years (0.017, 95% CI −0.041–0.008, *p* = 0.187).

## 4. Discussion

The last few decades have seen a tremendous improvement in surgical treatment of neuromuscular spinal deformities. Adoption of segmental screw fixation, advanced osteotomy techniques, improved pelvis fixation methods, and more advanced anaesthetics have significantly broadened the scope and improved the outcomes of spinal surgery in CP patients [14]. Although several authors have confirmed the positive impact of deformity correction on HRQoL, surgery remains a big undertaking for patients and their families [11,12,15]. Children with CP have longer hospital stay, spend more time in ICU, often have multiple comorbidities, and have a higher complication rate. These factors have a significant impact on costs of surgery at patient, hospital, and society level [16,17].

CUA represents the current gold standard of health economics and is routinely used to make decisions about resources allocation [1]. To the best of our knowledge, no formal CUA for scoliosis surgery in CP has been attempted so far. The main reason for this is the lack of prospective utility scores collected through validated preference-based questionnaires. HUI3 is not always available, and at times can be cumbersome to collect in patients who cannot directly communicate with the examiner. Furthermore, because HUI3 has been developed at general population level it may lack some of the clinical endpoints that are specific to a particular subset of patients. As a result, none of the larger trials or case series analysing outcomes of scoliosis surgery in CP patients has reported preference-based HUI3 scores.

Our study shows that CPCHILD scores can be reliably converted into HUI3 utility values for CUA. The mapping algorithm includes three variables, namely CPCHILD total score, feeding, and communication ability. These last two variables are easy to collect in clinical practice and significantly improve predictive ability (i.e., goodness of fit) of the model. When used to predict HUI3 values at 1- and 2-years follow-up, the algorithm was able to provide accurate estimations within 0.020 points from real data. This is also below the reported threshold for minimal clinical difference (MCD) of HUI3 at 0.032 points [18].

**Figure 2:**
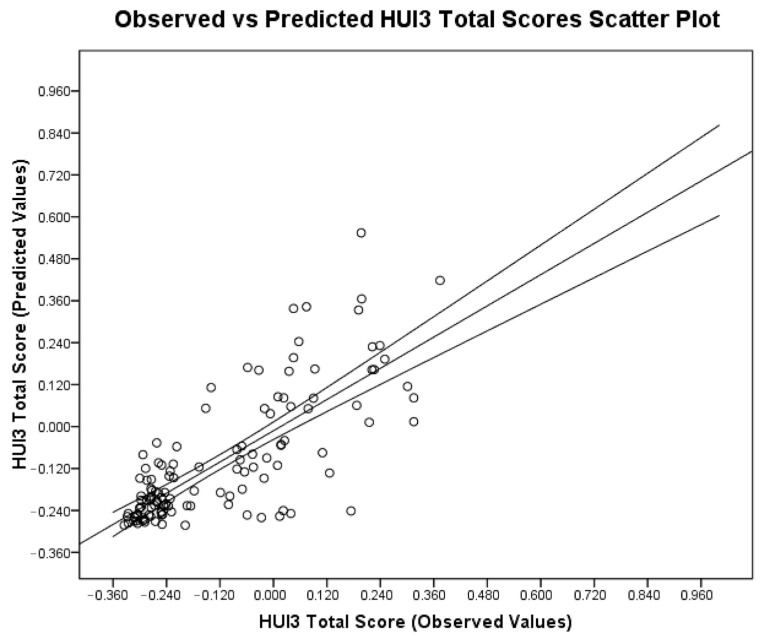
Observed vs. predicted HUI3 Total Utility Values scatterplot. The regression line with 95% confidence interval is shown in the graph. The graph shows that prediction algorithm is more accurate for lower HUI3 values (more heavily affected patients) than higher values.

Although a large population of CP patients has been used to develop the model, generalizing our results to less severely affected CP patients with minor spinal deformities may be questionable. More than 90% of our patients were GMFCS IV and V, more than 85% of them underwent surgery during the study period, and the average magnitude of scoliosis deformity was 81.93° ± 25.13° pre-operatively. This explains why a left skewed distribution of HUI3 scores (i.e., lower scores ≤ 0.100) was observed in our cohort. (Figure 1) To account for this a logarithmic transform was introduced in the linear regression equation, resulting in an exponential line fit. This allows a better predictive ability for HUI3 scores at the lower end of the range (i.e., more severely affected patients), but progressively larger 95% confidence intervals for higher scores. (Figure 2).

Our study has some limitations. The lack of an external validation dataset with both CPCHILD and HUI3 data available does not allow assessment of external validity of the mapping model. This is an important property of any mapping algorithm and was not assessed in the present study. Nevertheless, we were able to confirm longitudinal validity of the algorithm by comparing observed and predicted HUI3 scores at 1- and 2-year follow-up (Table 6). This is an important property for CUA evaluation where outcomes of different treatments are compared over time. Notably, roughly 40% of the variance of the observed HUI3 scores cannot be explained by the model. This is expected considering the different development strategy and aims of the CPCHILD and HUI3 questionnaires. As such, our model cannot be considered a replacement of the HUI3 questionnaire. “Survey-fatigue” or “burn-out” may have introduced some degree of bias given multiple questionnaires (CPCHILD and HUI3) were filled out at each time point.

In addition, it is important to note that the CPCHILD and HU13 questionnaires were completed by either the patient’s parents or equivalent primary caregivers at all time points. This in turn may not fully reflect the actual health utility experience of the child and may have parent/care-giver bias. The study nonetheless still provides valuable information given that the HRQoL and Health Utility indices were collected prospectively on all patients by the same parent/caregiver at all time points.

The existing evidence on the effectiveness of scoliosis surgery in patients with severe CP cannot be used for economic evaluations as the HSUV have all been generated from instruments based upon surveys from the general population using non-preference questionnaires. The use of mapping algorithms to generate health-state utility values where there is no preference-based measure has become a common strategy for economic appraisal of health-care interventions. The preferred mapping algorithms identified in this analysis will be of value for cost-utility analyses for CP patients undergoing spinal deformity surgery. Cost-utility assessment of spinal deformity surgery in CP patients can now be addressed and compared in clinical trials that lack a preference-based HRQoL measure.

## Figures and Tables

**Table 1:** Patient sample demographics (*n* = 232).

Age at enrolment (years)	14.05 ± 2.60 (8.25–20.92)	
Sex (%)	M 54.3%, F 45.7%	
Weight (Kg)	33.61 ± 9.17 (16.00–81.00)	
GMFCS level (%)	I II III IV V	(0.5%) (3.2%) (4.5%) (17.2%) (74.7%)
Develop. delay (%)	None Mild Moderate Severe Profound	(5.3%) (8.4%) (9.3%) (40.1%) (37.0%)
Communication (%)	Verbal Limited Non-verbal	(13.9%) (13.5%) (72.6%)
Seizures (%)	None Yes, controlled Yes, not controlled	(31.9%) (53.4%) (14.7%)
Feeding (%)	Oral Oral and G-tube G/J-tube only	(47.0%) (16.1%) (37.0%)
Coronal Cobb angle (°)	81.93 ± 25.13 (8–143)	
Cobb < 75° (%)	43.1%	
Cobb ≥ 75° (%)	56.9%	
T2-T12 kyphosis (°)	41.22 ± 24.03 (−43.00–107.00)	
T5-T12 kyphosis (°)	35.85 ± 23.51 (−35.00–109.00)	
T12-S1 lordosis (°)	38.15 ± 31.56 (−48.00–120.00)	
Pelvic obliquity (°)	25.40 ± 15.72 (0.00–62.00)	

Data expressed as mean ± standard deviation (range), percentages are used where appropriate.

**Table 2:** CPCHILD^TM^ Scores at enrolment (*n* = 232).

	CPCHILD Personal Care/Daily Living	CPCHILD Positioning, Transferring & Mobility	CPCHILD Comfort & Emotions	CPCHILD Communication & Social Interaction	CPCHILD Health	CPCHILD Quality of Life	CPCHILD Total
Score	38.47 ± 16.83	34.53 ± 17.94	75.64 ± 20.96	54.03 ± 28.67	58.17 ± 18.70	63.19 ± 24.52	51.87 ± 14.56
Range	(0.00–85.19)	(0.00–91.67)	(16.33–100.00)	(0.00–100.00)	(20.00–100.00)	(0.00–100.00)	(17.88–90.03)
Ceiling (*n*, %)	0 (0.0%)	0 (0.0%)	13 (5.6%)	16 (6.9%)	4 (1.7%)	27 (11.6%)	0 (0.0%)
Floor (*n*, %)	8 (3.4%)	4 (1.7%)	0 (0.0%)	6 (2.6%)	0 (0.0%)	7 (3.0%)	0 (0.0%)

Data are expressed as mean ± standard deviation (range) unless stated otherwise.

**Table 3:** HUI3 Scores at enrolment (*n* = 232).

	HUI3 Ambulation	HUI3 Cognition	HUI3 Dexterity	HUI3 Emotion	HUI3 Hearing	HUI3 Pain	HUI3 Speech	HUI3 Vision	HUI3 Total
Score	0.622 ± 0.076	0.628 ± 0.252	0.659 ± 0.147	0.962 ± 0.071	0.980 ± 0.085	0.923 ± 0.084	0.790 ± 0.129	0.867 ± 0.155	−0.096 ± 0.222
Range	(0.580–1.000)	(0.420–1.000)	(0.560–1.000)	(0.640–1.000)	(0.400–1.000)	(0.550–1.000)	(0.680–1.000)	(0.610–1.000)	(−0.335–0.945)
Ceiling (*n*, %)	3 (1.3%)	34 (14.6%)	15 (6.5%)	125 (53.9%)	201 (86.6%)	50 (21.5%)	31 (13.4%)	76 (32.7%)	0 (0.0%)
Floor (*n*, %)	0 (0.0%)	0 (0.0%)	0 (0.0%)	0 (0.0%)	0 (0.0%)	0 (0.0%)	0 (0.0%)	0 (0.0%)	0 (0.0%)

Data are expressed as mean ± standard deviation (range) unless stated otherwise.

**Table 4:** Spearman’s correlation coefficients for paired observations between CPCHILD and HUI3 questionnaires.

CPCHILD Subdomains	HUI3 Attributes								
	Ambulation	Cognition	Dexterity	Emotion	Hearing	Pain	Speech	Vision	Total
Personal	0.021	0.051	0.038	0.121	0.071	0.276 **	−0.001	−0.030	0.053
Position	0.174 *	0.315 **	0.106	0.144	−0.031	0.355 **	0.139	0.044	0.264 **
Comfort	0.191 *	0.073	0.152 *	0.452 **	0.139	0.644 **	0.034	0.121	0.204 *
Communication	0.223 **	0.585 **	0.385 *	0.356 **	0.103	0.261 **	0.556 **	0.369 **	0.646 **
Health	0.116	0.312 **	0.243 **	0.244 **	0.104	0.251 **	0.316 **	0.151 *	0.378 **
QoL	0.162 *	0.169 *	0.086	0.437 **	0.051	0.388 **	0.097	0.127	0.242 **
Total	0.224 **	0.367 **	0.254 **	0.402 **	0.087	0.530 **	0.296 **	0.242 **	0.459 **

* Correlation is significant at the 0.05 level (2-tailed); ** Correlation is significant at the 0.01 level (2-tailed).

**Table 5:** Regression model for HUI3 prediction.

Model	β	*p*		
A (Constant)	−1.700		R^2^	0.558
CPCHILD Total	0.016	0.0001	Adj R^2^	0.547
Communication	−0.436	0.0001	RMSE	0.578
Feeding	−0.289	0.0001	F	49.73 (*p* = 0.0001)
Model: ln(HUI3) = −1.700 + (0.016 × CPCHILD Total) − (0.436 × Communication ^†^) − (0.289 × Feeding ^‡^)				

^†^ Communication: Verbal = 0, Limited verbal = 1, Non-verbal = 2; ^‡^ Feeding: Oral only = 0, Oral and G-tube = 1, G-tube only = 2.

**Table 6:** Comparison of observed and estimated HUI3 scores (model testing).

	Mean	SD
1 yr follow-up data		
HUI3 observed values	−0.112	0.218
HUI3 calculated values	−0.092	0.197
Mean difference (95% CI)	−0.020 (−0.045–0.006)	0.153
*p* value ^†^	0.129	
2 yr follow-up data		
HUI3 observed values	−0.104	0.213
HUI3 calculated values	−0.087	0.219
Mean difference (95% CI)	−0.017 (−0.041–0.008)	0.151
*p* value ^†^	0.187	

^†^ *p* values from paired *t*-test.

## Data Availability

The data presented in this study are available on request from the corresponding author due to privacy reasons.
